# Geopolymer: A Systematic Review of Methodologies

**DOI:** 10.3390/ma15196852

**Published:** 2022-10-02

**Authors:** Jabulani Matsimbe, Megersa Dinka, David Olukanni, Innocent Musonda

**Affiliations:** 1 Department of Civil Engineering Science, Faculty of Engineering and the Built Environment, University of Johannesburg, Johannesburg 2006, South Africa; 2 Centre for Applied Research and Innovation in the Built Environment (CARINBE), Faculty of Engineering and the Built Environment, University of Johannesburg, Johannesburg 2092, South Africa; 3 Department of Mining Engineering, Malawi University of Business and Applied Sciences, P/Bag 303, Chichiri, Blantyre 3, Malawi; 4 Department of Civil Engineering, Covenant University, 10 Idiroko Road, Ota 112104, Ogun State, Nigeria

**Keywords:** geopolymer, geopolymer mortar, geopolymer concrete, inorganic polymers, alkali-activated materials

## Abstract

The geopolymer concept has gained wide international attention during the last two decades and is now seen as a potential alternative to ordinary Portland cement; however, before full implementation in the national and international standards, the geopolymer concept requires clarity on the commonly used definitions and mix design methodologies. The lack of a common definition and methodology has led to inconsistency and confusion across disciplines. This review aims to clarify the most existing geopolymer definitions and the diverse procedures on geopolymer methodologies to attain a good understanding of both the unary and binary geopolymer systems. This review puts into perspective the most crucial facets to facilitate the sustainable development and adoption of geopolymer design standards. A systematic review protocol was developed based on the Preferred Reporting of Items for Systematic Reviews and Meta-Analyses (PRISMA) checklist and applied to the Scopus database to retrieve articles. Geopolymer is a product of a polycondensation reaction that yields a three-dimensional tecto-aluminosilicate matrix. Compared to unary geopolymer systems, binary geopolymer systems contain complex hydrated gel structures and polymerized networks that influence workability, strength, and durability. The optimum utilization of high calcium industrial by-products such as ground granulated blast furnace slag, Class-C fly ash, and phosphogypsum in unary or binary geopolymer systems give C-S-H or C-A-S-H gels with dense polymerized networks that enhance strength gains and setting times. As there is no geopolymer mix design standard, most geopolymer mix designs apply the trial-and-error approach, and a few apply the Taguchi approach, particle packing fraction method, and response surface methodology. The adopted mix designs require the optimization of certain mixture variables whilst keeping constant other nominal material factors. The production of NaOH gives less CO_2_ emission compared to Na_2_SiO_3,_ which requires higher calcination temperatures for Na_2_CO_3_ and SiO_2._ However, their usage is considered unsustainable due to their caustic nature, high energy demand, and cost. Besides the blending of fly ash with other industrial by-products, phosphogypsum also has the potential for use as an ingredient in blended geopolymer systems. The parameters identified in this review can help foster the robust adoption of geopolymer as a potential “go-to” alternative to ordinary Portland cement for construction. Furthermore, the proposed future research areas will help address the various innovation gaps observed in current literature with a view of the environment and society.

## 1. Introduction

Geopolymer is no longer viewed as a concept for a greener society but rather as a pragmatic solution for the reduction in CO_2_ emissions in the construction and building sector. The utilization of geopolymer as a potential cement alternative in construction has been shown to reduce the overall CO_2_ emission by 9% to 64% [1,2], depending on geographic location, transport scenarios, activator production process, and mix design. The contribution of CO_2_ emission to global warming stands at 65%, and the production of one tonne of ordinary Portland cement (OPC) emits one tonne of CO_2_ into the atmosphere [3,4]. According to [5], geopolymer offers a strong reduction in global warming by releasing 169 kg CO_2_/m^3^ while ordinary Portland cement releases 306 kg CO_2_/m^3^ for the same mechanical properties, representing a decrease in emission by 45%. The increased awareness of sustainability and global warming concerns has urged the construction industry to research greener alternative cement to OPC [6,7]. Sustainability is considered in terms of a cost/benefit ratio [8]. The global construction industry contributes 13.5% to the global gross domestic product [9] and is essential for advancement in modern civilization. Greater than 90% of the CO_2_ in a concrete mixture is from the binder; therefore, controlling the type and amount of binder can control sustainability [8]. The growing number of geopolymer pilot projects [10,11,12] and the global research interest in the area [13,14,15,16,17] highlight the increasing attention given to geopolymer technology. Implementation goals in line with alternative cementitious materials such as geopolymers are discussed and proposed at the international level, e.g., the International Energy Agency Global Roadmap for Buildings and Construction of 2020–2050 and the UN Climate Change Conference of the Parties (COP21) Paris Agreement [18]. 

Conventional concrete production requires a mixture of ordinary Portland cement, water, and aggregates. Ordinary Portland cement is second to water in terms of global utilization and contributes 5–8% of total global CO_2_ emissions [4,19,20,21]. Besides steel and aluminum products, ordinary Portland cement is the third most energy-intensive material consuming 7% of the total energy utilized by industries worldwide [22]. To comply with the COP21 Paris Agreement of limiting global warming to below 2 °C by 2030 and net-zero by 2050 [18], the research and development of geopolymers in America, Europe, Asia, and Australia have grown significantly whilst very limited research has been performed in Africa [23]. With reference to the Scopus database, the African countries leading in geopolymer research consist of Egypt, Cameroon, Nigeria, and Morocco hence the need to scale up the research in alternative geopolymer cement for infrastructural development in Africa. The use of geopolymer is considered a potential new alternative binder [3,24] with lower CO_2_ emission [25]. Geopolymer is a chemical reaction product from an aluminosilicate precursor and alkaline or acidic activating solution. It is usually produced as a geopolymeric binder, mortar, concrete, brick, porous media, etc. An increase in industrial waste and landfills is directly proportional to the growth in industrialization and the global population. For example, as the need for steel, energy, and fertilizer increases, the volume of industrial waste materials such as ground granulated blast furnace slag (GGBFS), coal fly ash (CFA), and phosphogypsum (PG) increase, respectively. Disposal of industrial waste materials in open dumpsites leads to detrimental environmental issues consisting of groundwater contamination, bulk storage spillage, and heavy metal leaching [26,27,28]. Therefore, the utilization of industrial by-products and waste materials for geopolymer production is considered economically and environmentally beneficial due to the increased need for green building materials [29,30,31,32]. However, geopolymer is still a new technology with gaps in design standards requiring further research and experiments to standardize it [33,34]. The standards that are currently in use for geopolymer production were designed for OPC and this might contribute to the resistance of the construction industry and regulatory authorities to fully implement geopolymer usage for infrastructure development. Due to the absence of geopolymer design standards, researchers have strived to contribute to the research field by proposing various definitions and mix design procedures to facilitate the adoption of this innovative material. The common factors limiting the adoption and acceptance of geopolymer consist of the variability in material properties of precursor [33], high temperature curing conditions [35], high energy demand for producing activating solution [35], high cost of activator [36], the aggressiveness of alkaline and acidic activating solution [36], alkali attack [37], and alkali-silica reaction [38].

Although several authors have carried out different reviews on geopolymer [2,6,7,30,36,39,40,41,42,43,44], there is a gap in comprehensive reviews clarifying geopolymer definitions and mix design methodologies. Just as OPC has been fully researched for over 170 years leading to robust design and durability standards, the state-of-the-art on geopolymer innovation is lagging and still needs further comprehensive reviews to gain clarity and guide the research community on design parameters. Further, also missing in the previous literature reviews are discussions on the performance of blended phosphogypsum geopolymers. Nodehi and Taghvaee [7] reviewed the common precursors and activators used in alkali-activated materials as well as their durability factors. They concluded that the variation in mechanical properties of alkali-activated materials is majorly influenced by the total molar ratio (Si/Al), alkali concentration, and ratio of used activators. A review by [2] focused more on solid waste management and the heavy-duty performance of waste-based geopolymer concrete in civil engineering, military, and road applications. A similar review focusing on geopolymer concrete applications was performed by [30]. Both [2,7] did not comprehensively expound on the most apparent factors affecting the behavior of waste-based geopolymers. There is a lack of clarity on definitions and mix design methods due to the varying scientific approaches to geopolymers [45] and alkali-activated materials [46]. The present review provides a comprehensive systematic analysis of geopolymer focusing on the definition and chemistry, precursor materials, activator type/concentration, admixture/additive, mix design, impact of curing, mechanical properties, microstructure, durability, and cost to clarify its viability as an alternative cement to ordinary Portland cement.

## 2. Research Significance

As earlier alluded to, the geopolymer concept is the future national and international alternative cement agenda. The abundant industrial by-products and waste materials should be fully utilized for geopolymer production to minimize the environmental impact and cost associated with its disposal in landfills. However, to progress this agenda, there is a need for clarity on the definitions and mix design methodologies for geopolymer. This review puts into perspective the most crucial facets to attain a good understanding of the unary and binary geopolymer design systems. The facets comprehensively reviewed in this investigation consisted of the definition and chemistry, raw materials, alkaline activator, impact of curing, admixture/additive, microstructure development, mechanical properties, mix design, durability, and cost. The various unary and binary geopolymer systems incorporating phosphogypsum are novelty areas also discussed in this review. Compared to unary geopolymer systems, binary geopolymer systems contain complex hydrated gel structures and mix designs that influence setting time, workability, strength, and durability. Lastly, this review highlights the practical applications, limitations, and future research areas to foster further innovation of geopolymer design methods.

## 3. Methodology

**Figure 1:**
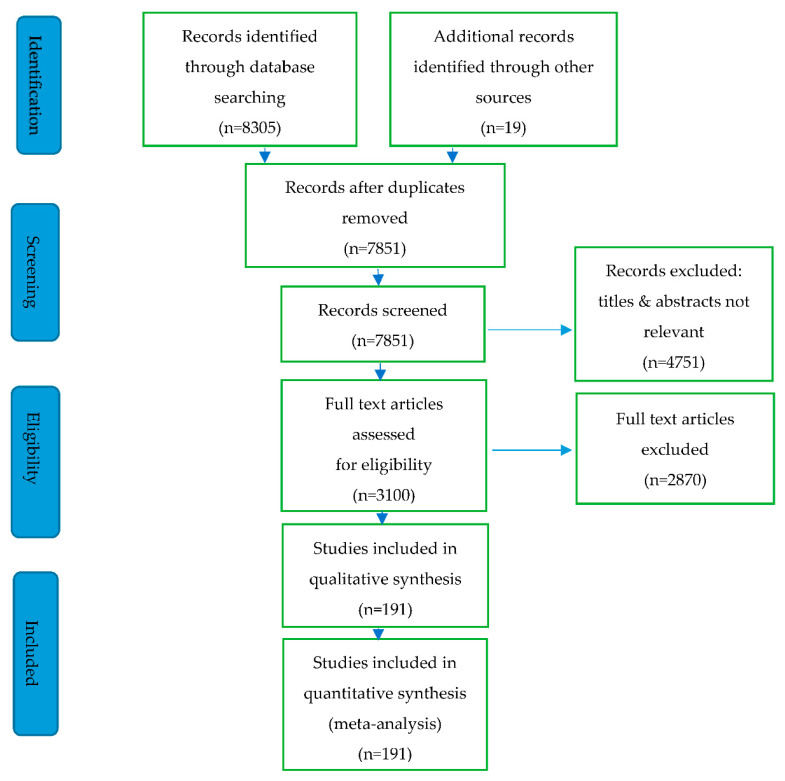
PRISMA flowchart for the study.

The methodology for this review is based on the scoping review protocol drawn using the Preferred Reporting of Items for Systematic Reviews and Meta-Analyses Protocol (PRISMA-P) checklist [47], as well as the Preferred Reporting of Items for Systematic Reviews and Meta-Analyses extension for Scoping Reviews (PRISMA-ScR) checklist [48]. Google Scholar, Web of Science, and Scopus are the most used publication databases [49,50]. Scopus is the most preferred due to its wider coverage of bibliometric data [49,50,51]. Authors [52,53] conducted their systematic literature reviews on construction materials using the Scopus database; therefore, the Scopus database was selected as a data retrieval tool for this review. An inclusion/exclusion criterion was developed to filter out irrelevant journal articles to the present investigation. The eligibility criteria, information sources, and search strategies were defined. The search strings defined to conduct the research were “geopolymer”, “geopolymer mortar”, “geopolymer concrete”, and “alkali-activated materials”. Table 1 shows the inclusion criteria applied to filter out unnecessary journal articles/reviews. After clarifying the main inclusion criteria, all the references of the selected articles were subjected to the snowballing technique [54] to gather additional relevant information. Figure 1 shows the PRISMA flowchart, where 191 articles were included for qualitative and quantitative synthesis. The risk of bias in this review was assessed using the ROBIS tool [55,56].

## 4. Results and Discussion

This section presents the systematic literature review on key parameters and clusters identified requiring further attention before the full adoption of geopolymers at the level of ordinary Portland cement [34,57,58,59]. The clusters are briefly addressed in the following subsections.

### 4.1. Definition and Chemistry

**Figure 2:**
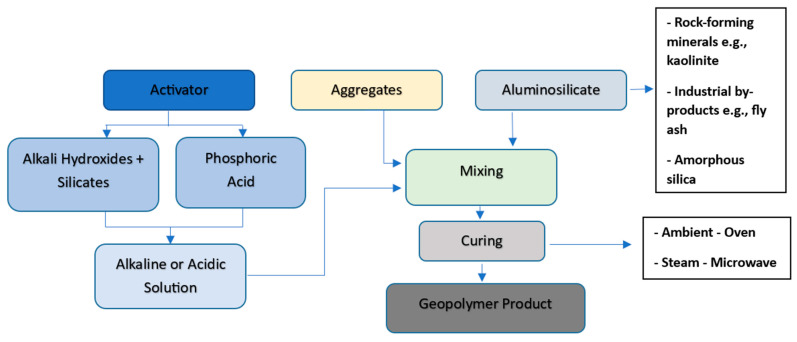
An overview of the geopolymer production process.

The term ‘geopolymer’ was coined in the 1970s by the French scientist and engineer Prof Davidovits and applied to a class of solid materials synthesized by the reaction of an aluminosilicate powder with an alkaline solution [3,60]. Aluminosilicate binders or inorganic polymeric compounds result from a polycondensation reaction that yields a three-dimensional tecto-aluminosilicate matrix known as polysialate having an empirical formula [3]: Mn[-(SiO_2_)z-AlO_2_]n.wH_2_O, where M is a cation (K^+^, Na^+^, Ca^2+^); n is the polycondensation degree, and z is 1,2, 3 or greater. Sialate refers to a ‘silicon-oxo-aluminate’ building unit whose network consists of SiO_4_ and AlO_4_ tetrahedra linked by sharing all oxygen atoms. The presence of the cations balances the remaining anions of the four-coordinated Si^4+^ and Al^3+^ ions. The term ‘Geopolymer’ describes the inorganic aluminosilicate polymer products from the reaction of amorphous aluminosilicate-containing materials and alkaline or acidic solution [3]. Figure 2 shows the various ingredients and the production process for geopolymer. The synthesis route is either an alkaline medium consisting of hydroxides and silicates or an acidic medium consisting of phosphoric acid. The various synthesis routes are mixed and reacted with an aluminosilicate precursor to commence the polymerization process. The aluminosilicate precursor can either be naturally occurring, such as kaolinite, or an industrial by-product, such as fly ash. The alkaline medium is the commonly used synthesis route since it is less caustic and hazardous than the acidic medium. Curing helps to regulate the polymerization process where strength is gained the slowest under ambient temperature.

**Figure 3:**
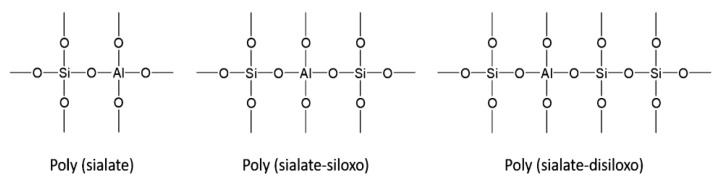
Main polymer chains of geopolymers [61].

As illustrated in Figure 3, this mineral chemical compound consists of repeating units [3], such as siloxo (-Si-O-Si-O-), sialate (-Si-O-Al-O-), sialate-disiloxo (Si-O-Al-O-Si-O-Si-O), ferro-sialate (-Fe-O-Si-O-Al-O-), or alumino-phospho (-Al-O-P-O-), created through a process of geopolymerization. Geopolymerization combines many small molecules known as oligomers into a covalently bonded network at low temperatures, below 100 °C, or room temperature [3].

**Figure 4:**
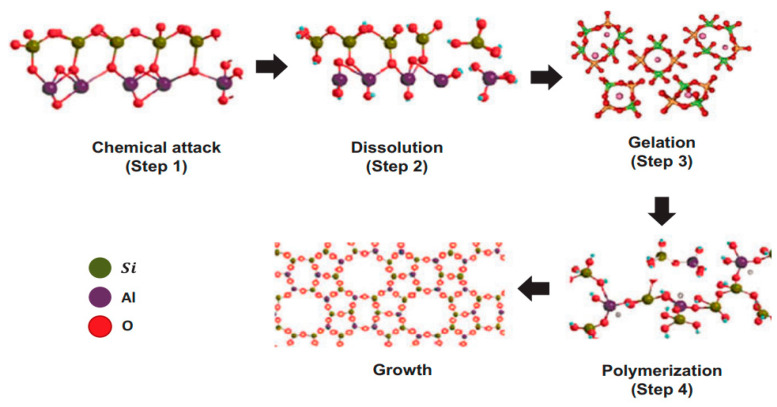
Mechanism of geopolymerization reaction [61].

Figure 4 depicts the geopolymerization process which involves (i) destruction–coagulation, (ii) coagulation–condensation, and (iii) condensation–crystallization [61]. The geochemical syntheses are performed through oligomers (dimer, trimer, tetramer, pentamer) which provide the actual unit structures of the three-dimensional macromolecular edifice. The geopolymer microstructure depends on temperature, where it becomes amorphous at ambient temperature and crystalline at temperatures above 500 °C and 1000 °C for sodium-based and potassium-based alkaline medium, respectively. Geopolymers are considered a new material, binder, or cement [24,62]. Other names in literature comprising alkali-bounded ceramics, alkali-activated cement, inorganic polymer, hydroceramic, and geocement are used to identify materials produced through a similar chemical process [46].

**Figure 5:**
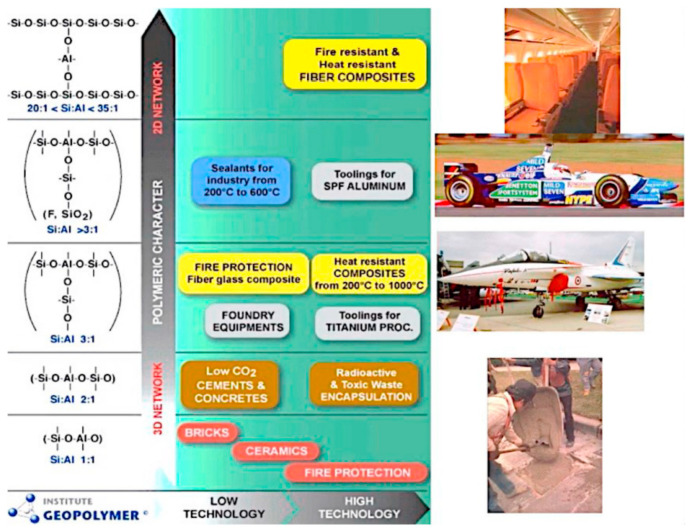
Applications of geopolymers with respect to polymeric chain [45].

Figure 5 shows the correlation between the polymeric network chain and field application. Single poly(sialate) (Si:Al 1:1) are applied in ceramics fabrication; poly(sialate-siloxo) (Si:Al 2:1) are applied in cement and concrete fabrication as well as toxic waste encapsulation; poly(sialate-disiloxo) (Si:Al 3:1) are applied in fire protection and heat resistant composites; sialate link (Si:Al > 3:1) are applied in sealants and toolings [3].

### 4.2. Raw Materials

Due to the high generation of industrial waste by-products, disposal concerns, less utilization, and hazardous nature, the research on its valorization as a precursor for geopolymer production is potentially environmentally viable. This review focused on the commonly available industrial by-products and/or waste materials utilized as aluminosilicate precursors in geopolymer production due to the global need for a circular economy and cradle-to-cradle life cycle [7,63,64]. Several researchers have exploited and extensively reviewed the potential utilization of natural raw materials as precursors in geopolymer production, comprising Kaolin [65,66,67], Metakaolin [31,68,69,70], Zeolite [71,72,73], Laterite [74,75,76], and Volcanic ash [77,78,79], which are outside the scope of this review and are therefore not discussed in the text that follows. The industrial by-products and/or waste as raw materials for geopolymer production are discussed below.

#### 4.2.1. Fly Ash

**Figure 6:**
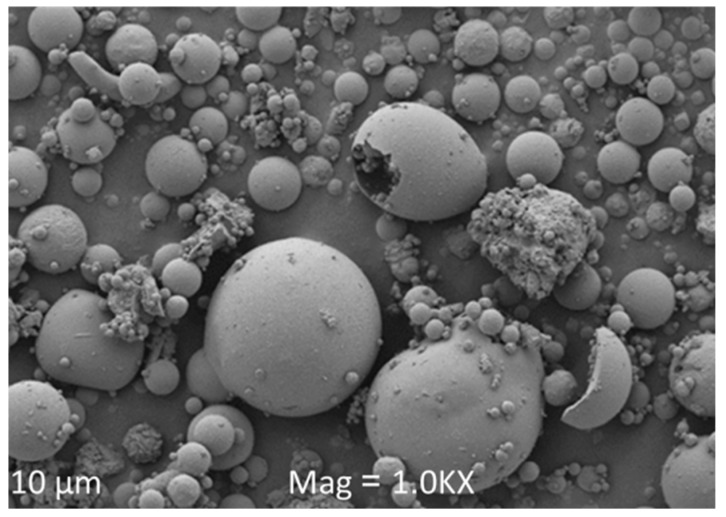
Morphology of fly ash [88].

Fly ash is a major industrial fine particulate by-product formed from the combustion of coal and captured by electrostatic precipitators in thermal power plants. Coal fly ash annual production rates for major coal-consuming countries indicate approximately 600 million tonnes for China [80], 240 million tonnes for India [81], 32 million tonnes for the United States of America [82], 13 million tonnes for Australia [41], and 40 million tonnes for South Africa [83]. Fly ash is used in different applications or disposed of worldwide [84]. Due to its abundance, fly ash is one of the commonly used aluminosilicate sources used in geopolymer manufacturing [85,86,87]. Table 2 gives the chemical composition of fly ash used in various parts of the world. The major compositions of the fly ash are SiO_2_, Al_2_O_3_, Fe_2_O_3_, and CaO, while the minor constituents are MgO, P_2_O_5_, K_2_O, Na_2_O, SO_3_, MnO, and TiO_2_. The fly ash is classified as low calcium Class F, i.e., siliceous ash with pozzolanic properties as per ASTM C618, which specifies that the sum of SiO_2_ + Al_2_O_3_ + Fe_2_O_3_ greater than 70 wt.% implies Class F and if the sum of SiO_2_ + Al_2_O_3_ + Fe_2_O_3_ is between 50 wt.% and 70 wt.% implies Class C. In addition, ASTM C618 specifies that if the CaO content is less than 10 wt.%, the fly ash is classified as low calcium Class F, usually from bituminous coal. The SiO_2_/Al_2_O_3_ ratio predicts the potential reactivity of ash as a supplementary cementitious material. The loss of ignition (LOI) of the fly ash samples is less than the LOI limit of 6% specified by ASTM C618 and this implies that the quantity of unburnt carbon in the ash and the performance of the boiler in the power plant are acceptable. Figure 6 shows the morphology of fly ash which is a spherical smooth surface with relatively small particles [88] observed under a scanning electron microscope.

Naghizadeh et al. [89] studied the behavior of fly ash geopolymer binders exposed to various alkaline media. The study used low calcium Class F South African fly ash as the main precursor and mixed it with sodium silicate and 12 M sodium hydroxide to prepare the geopolymer binder. The mortar mixture contained a 2.25 aggregate/binder ratio, alkali activator solution of SiO_2_/Na_2_O = 1.4, and activator/FA ratio = 0.5. The 14 days’ compressive strength at 80 °C curing temperature of the non-immersed, water immersed, 1 M NaOH mortar, and 3 M NaOH immersed mortar were 53.2 MPa, 50.6 MPa, 48.9 MPa, and 14.6 MPa, respectively. The decrease in the strength was attributed to the dissolution of Si and Al from the aluminosilicate gel network under an alkali attack. These results showed that severe alkali attack on fly ash-based geopolymer binder only occurs at alkali concentrations equal to/greater than 3 M NaOH. In another study, Rajmohan et al. [86] reported the mechanical and durability performance of heat-cured low calcium fly ash-based sustainable geopolymer concrete. In the study, the 28-day compressive strength before and after exposure to sulfate solution at 60 °C curing temperature was 56.18 MPa and 42.58 MPa; at 80 °C was 55.46 MPa and 44.92 MPa; at 100 °C was 50.79 MPa and 41.46 MPa, respectively. These results demonstrate that the fly ash-based geopolymer concrete gives adequate compressive strength and improved resistance to sulfate and acid attacks.

#### 4.2.2. Phosphogypsum

**Figure 7:**
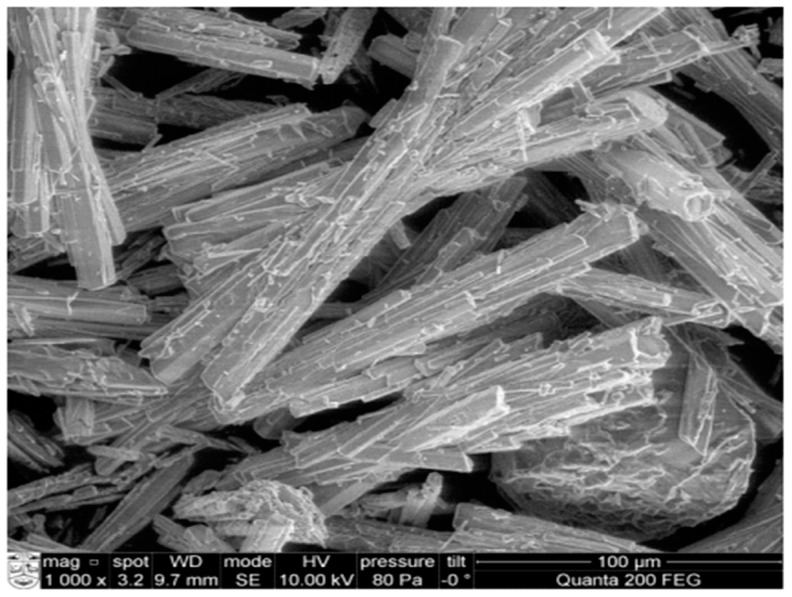
Morphology of phosphogypsum [98].

Phosphogypsum (PG) is an industrial by-product of the wet-process production of phosphoric acid through the reaction of phosphate ore and sulphuric acid. PG annual production rates for major fertilizer manufacturing countries indicate approximately 50 million tonnes for China [93], 11 million tonnes for India [94], 40 million tonnes for the United States of America [95], and 35 million tonnes for South Africa [96]. Due to its abundance, other researchers have reused PG as a construction/building material [26,39,97], and soil stabilization material [96]. Table 3 gives the chemical composition of phosphogypsum used in various parts of the world. The major compositions of the PG are CaO and SO_3_. Figure 7 shows the morphology of phosphogypsum which is of dense crystalline structure and irregularly shaped parallelepipeds [98] observed under a scanning electron microscope.

Hua et al. [93] studied the effects of fibers on mechanical properties and freeze–thaw resistance of phosphogypsum-slag-based geopolymer and found that the addition of 1% fiber increased the flexural strength from 7.9 MPa and 8.4 MPa (0% fiber) to 9.5 MPa and 12.5 MPa at 7 and 28 days, respectively. The alkaline activator was prepared using sodium silicate (water glass) of modulus 3.0 and sodium hydroxide. The pastes were made with a water/powder ratio of 0.6. The results indicated in the study showed that polypropylene fiber provides a phosphogypsum-slag mix with better performance than mineral and glass fibers. In another study, Ref. [99] reported the x-ray diffraction and scanning electron microscopy of fly ash-phosphogypsum geopolymer bricks using 10 M sodium hydroxide and sodium silicate. For the 70–75 °C oven-cured samples for 12 h, the compressive strength was 20.31 MPa (9% PG content), 23.13 MPa (13% PG content), 23.06 MPa (14% PG content), and 15.13 MPa (25% PG content) showing that an increase in PG content beyond 13% lead to a decrease in compressive strength. For the 24 h air-cured samples, the compressive strength was 18.31 MPa (9% PG content), 21.38 MPa (13% PG content), 21.37 MPa (14% PG content), and 13.92 MPa (25% PG content) showing that an increase in PG content beyond 13% lead to a decrease in compressive strength. The results showed that the FA-PG geopolymer bricks with higher compressive strength can be used for building applications, whilst those with lower compressive strength can be used as filler material. Rashad [100] studied the potential use of calcined phosphogypsum (CPG) (heated at 850 °C for 2 h) in alkali-activated fly ash (FA) under the effects of elevated temperatures and thermal shock cycles. The 28 days compressive strength of the FA/CPG mix proportions of 100/0, 95/5, 90/10, and 85/15 using sodium silicate as alkali activator (density 1.38 g/cm^3^, 8.2% Na_2_O, 27% SiO_2_, 64% H_2_O) cured at 75 °C for 7 days then ambient cured for the extra 21 days was 14.95 MPa, 26 MPa, 23 MPa, and 12.5 MPa, respectively. The increase in compressive strength at 5 and 10% CPG inclusion is attributed to a reduction in apparent porosity and un-hydrated particles, giving a relatively dense and interlocking structure. The rapid decrease in compressive strength at 15% CPG inclusion is attributed to a flocculent and porous structure that gives euhedral prismatic crystals of gypsum, which act as a barrier against geopolymer chain formation and thus reducing the cohesion of the microstructure. The residual strength increased with increasing heat treatments. The results indicate the possibility of recycling phosphogypsum in an alkali-activated fly ash system such as an ordinary Portland cement system. In another study, Ref. [101] reported the production of geopolymer binders at room temperature using calcined clay, waste brick, PG (4%, 8%, 12%, and 16%), and sodium hydroxide alkali activator (10 M, 14 M, and 17 M) with a solid/liquid ratio of 3. The highest 28-day compressive strength of 36 MPa was achieved at 8% PG replacement and 14 M sodium hydroxide, after which the calcined clay was replaced by waste brick (WB) powder which achieved similar mechanical properties as the optimal condition. An increase in phosphogypsum content above 8% decreased the compressive strength due to the excess sulfate disturbing the geopolymer structure by forming ettringite which causes swelling through moisture absorption. The results foster the utilization of low-cost waste materials (PG and WB) for geopolymer binder production and further recommend studying the fire resistance, acid attack, chloride attack, sulfate attack, and permeability.

#### 4.2.3. Bottom Ash

**Figure 8:**
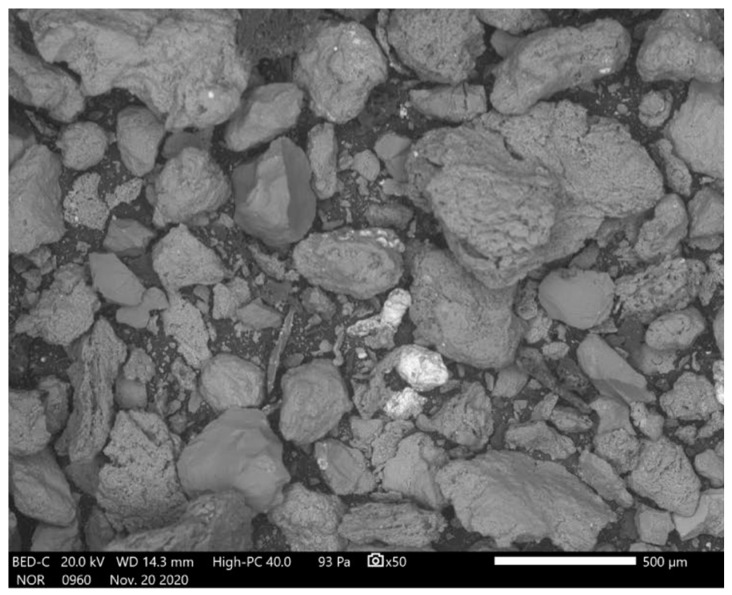
SEM image of bottom ash [102].

As compared to fly ash, bottom ash is collected at the bottom of the boiler and has coarser, angular, large-sized particles with a greater content of unburned carbon [102]. Figure 8 shows the SEM image of bottom ash which has coarser, irregular large-sized angular particles.

Despite its inferior properties, several geopolymer research incorporating bottom ash has been conducted. Ref. [103] studied the behavior of low-calcium fly and bottom ash-based geopolymer concrete cured at ambient temperature. In the study, the compressive strength of coal ash-based geopolymer concrete increased with a decrease in the liquid-to-binder ratio or an increase in the mass ratio of the fly ash-to-bottom ash. In another study, Ref. [104] reported the properties of cellular lightweight high calcium bottom ash-Portland cement geopolymer mortar. The results showed that the compressive strength increased with an increase in the binder and sodium hydroxide content but decreased with an increase in the foam content.

#### 4.2.4. Ground Granulated Blast Furnace Slag

**Figure 9:**
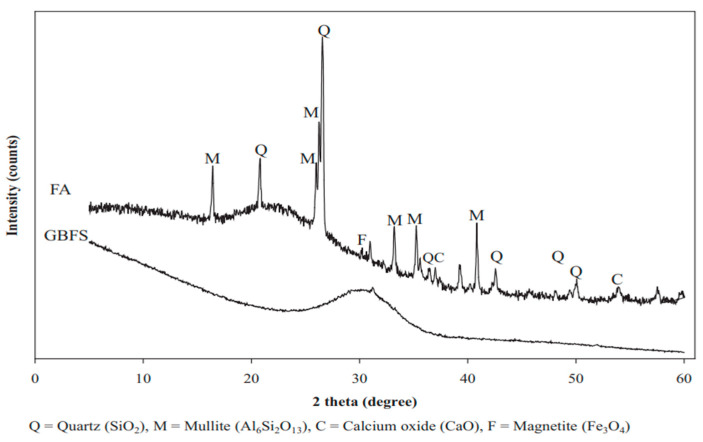
Comparative XRD of GGBFS and FA [105].

Ground granulated blast furnace slag (GGBFS) is a granular by-product material from the production of iron. Figure 9 shows that the XRD of GGBFS consists of an amorphous phase shown by a hump around 25–35°2 theta with a small amount of Fe_3_O_4,_ whilst the XRD of FA consist of an amorphous phase shown by a hump around 18–28°2 theta with some crystalline phases of Al_6_Si_2_O_13_, SiO_2_, magnesioferrite, and CaO [105]. Due to its glassy phase nature and calcium content, GGBFS is easier to activate through alkali activation and forms C-S-H or C-A-S-H gels as chemical reaction products which improve the geopolymer strength just as in OPC.

Calcium alumino-silicate hydrate (C-A-S-H) gel is the main reaction product during GGBFS activation, giving enhanced early strength development and reduced setting time in ambient curing conditions [106,107]. Aziz et al. [108] studied the strength development of solely GGBFS geopolymers and found that the 28 days compressive strength of 168.7 MPa was achieved with a solid/liquid ratio of 3.0, alkaline activator ratio of 2.5, and formation of tobermorite and calcite. Sithole et al. [109] studied the feasibility of synthesizing geopolymer bricks through alkaline activation of GGBFS and found that the 5 days compressive strength at 80 °C curing temperature, 15 M sodium hydroxide, and 0.15 liquid/solid ratio was 72 MPa attributed to the formation of a dense, less porous, and more amorphous microstructure. The geopolymer brick met the minimum compressive strength of 20.7 MPa, and water absorption of less than 17% as per ASTM C126-99 and ASTM C216-07a, respectively, for usage as facing and solid masonry brick. In another study, Ref. [110] reported the effect of GGBFS inclusion on the reactivity and microstructure properties of fly ash-based geopolymer concrete cured in ambient conditions. The 28-day compressive strengths of 30 MPa, 35 MPa, 40 MPa, and 45 MPa were achieved at 0%, 10%, 20%, and 30% replacement levels, respectively, attributed to the formation of calcium silicate hydrate (C-S-H) gel when increasing the GGBFS percentage levels.

#### 4.2.5. Basic Oxygen Furnace Slag

**Figure 10:**
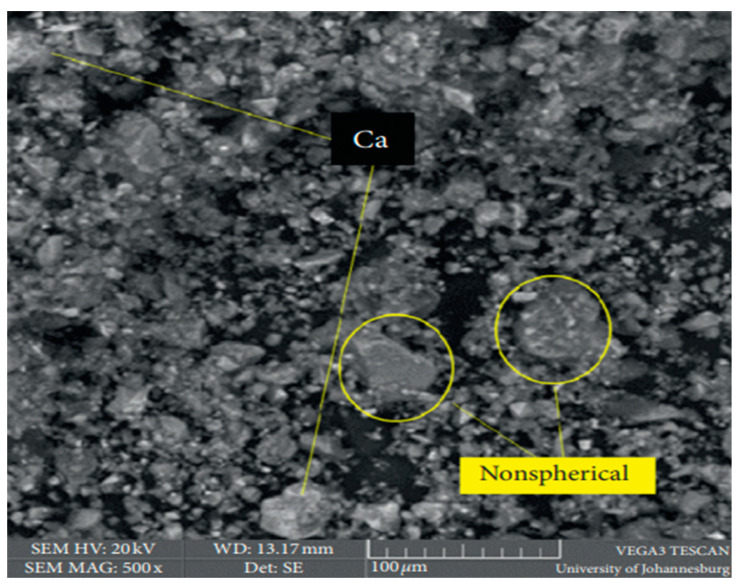
SEM image of BOFS [111].

Basic oxygen furnace slag (BOFS) is a by-product material from the production of steel. Figure 10 shows that the morphology of BOFS is composed of non-spherical glassy irregularly shaped microstructures. The observed rougher and cloudy texture surface is attributed to the presence of CaO [111].

Sithole et al. [112] studied the mechanical performance of fly ash modified BOFS-based geopolymer masonry bricks and concluded that to meet the ASTM C34-13 for masonry bricks, the most favorable conditions for the geopolymer synthesis were 10% fly ash, 5M sodium hydroxide, and 80 °C curing temperature. In another study, Mashifana et al. [111] explored the utilization of BOFS and gold mine tailings (GMT) for geopolymer synthesis and found that the 5 days compressive strength of the GMT: BOFS geopolymer cured at 90 °C was 20 MPa and 25.7 MPa for sodium hydroxide and potassium hydroxide alkaline activators, respectively, which satisfied the ASTM 34-17a and SANS 227:2007 for burnt masonry units, mine backfill paste, and lightweight civil works. Lee et al. [113] reported the stabilization of BOFS geopolymer and found that the 28-day compressive strength was 30–40 MPa, and its expansion was less than 0.5% after the ASTM C151 autoclave testing. The results show that slag can be turned into a value-added product through green geopolymer composites.

#### 4.2.6. Silica Fume

**Figure 11:**
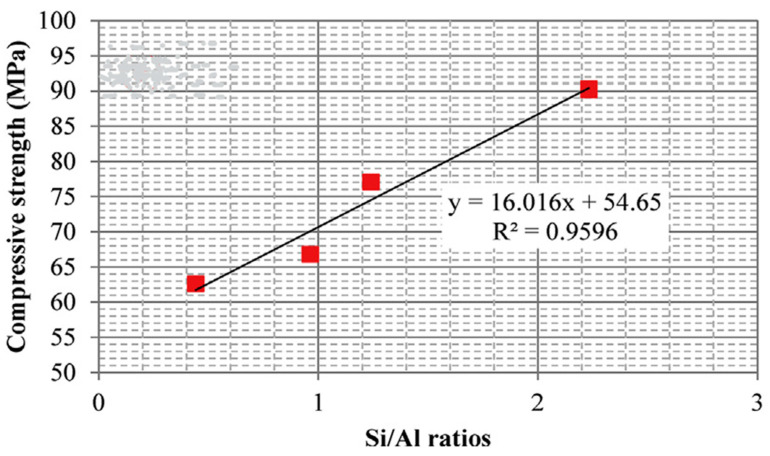
Compressive strength in the function of the Si/Al ratios [115].

Silica fume is an ultrafine by-product powder from the production of elemental silicon and ferrosilicon alloy in electric arc furnaces. Li et al. [114] studied the rheological and viscoelastic characterizations of fly ash/slag/silica fume-based geopolymer and concluded that the use of 20–30% silica fume to partially replace slag reduces the shear stress of fly ash-slag-based geopolymer grouting material. The results indicated that geopolymer instead of ordinary Portland cement paste can be used as a grouting material, and further recommend studying the shrinkage and creep of geopolymer grouting material. In another study, Duan et al. [115] observed that the inclusion of silica fume from 10–30% replacement levels increased the compressive strength of geopolymer, attributing it to the increase in chemical reaction products leading to a more dense, compact, and homogeneous microstructure. Similar results were observed by [116]. Figure 11 shows the correlation between the Si/Al ratio and the compressive strength, which is directly proportional. The dissolution of the silica fume in the blended mixture leads to a further increase in the Si/Al ratio in the aluminosilicate gel and thus increases the compressive strength.

#### 4.2.7. Flue gas desulphurization gypsum

Flue gas desulphurization gypsum (FGDG) is an industrial by-product produced through limestone-gypsum wet desulphurization of coal-fired flue gas with calcium sulfate dihydrate (CaSO_4_·2H_2_O) as the main product [117]. Guo et al. [118] studied the utilization of thermally treated FGDG and fly ash (FA) in geopolymer preparation and found that geopolymer containing 90% FA and 10% FGDG thermally treated at 800 °C for 1 h gave a better compressive strength of 37 MPa.

#### 4.2.8. Red Mud

Red mud (bauxite residue) is a by-product generated during bauxite ore processing into alumina through the Bayer process. Sun et al. [119] studied the mechanical and environmental characteristics of red mud geopolymers and found that the 28-day compressive strength of red mud geopolymers ranged from 35.2 MPa to 68.7 MPa. The leaching of heavy metal and trace elements met the thresholds of cementitious materials. In the study, it was recommended that future work should study the chemical states, the influence of pore structure, and the pore solution of red mud geopolymers. The mechanical properties of red mud geopolymers are affected by source material properties, alkaline activator concentration, and curing conditions [120,121].

#### 4.2.9. Mine Tailings

Falayi [122] reported the comparison between fly ash (FA) and basic oxygen furnace slag (BOFS) modified gold mine tailings (GMT) geopolymer and found that BOFS-GMT geopolymer and FA-GMT geopolymer gave compressive strengths of 21.44 MPa and 12.98 MPa cured at 70 °C and 90 °C, respectively. The higher strength in BOFS geopolymer was attributed to the formation of C-A-S-H gel.

#### 4.2.10. Rice Husk Ash

**Figure 12:**
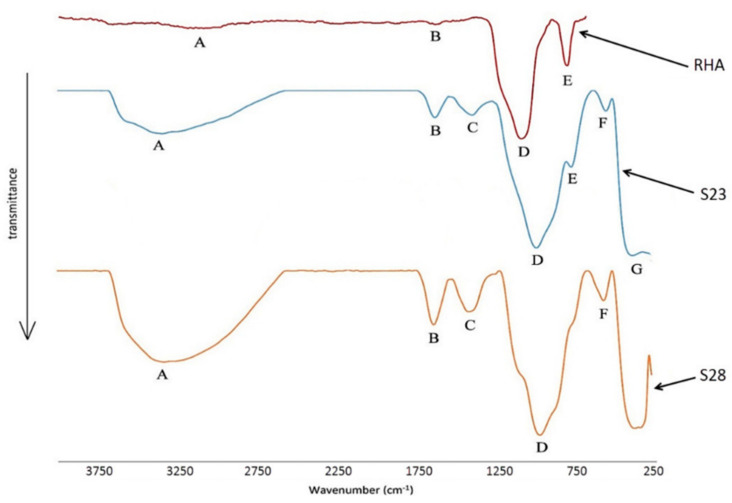
FTIR spectra of RHA [124].

Rice husk ash is a by-product of burning rice husk as a fuel source in boilers to generate electricity. Somna et al. [88] utilized rice husk ash (RHA) to produce RHA-FA geopolymer hollow block and concluded that the increase in RHA content increased the geopolymer compressive strength. In the study, the mix proportion of 50%FA:50%RHA with 14 M sodium hydroxide gave the preferred 28-day compressive strength of 8.5 MPa, which satisfied the TIS 58-2560 standard for usage in concrete hollow block manufacturing. Another study by [123] concluded that the use of RHA in geopolymer production is a sustainable and eco-friendly route for the construction industry. However, the sustainability and the eco-friendly route are questionable considering the caustic nature and high cost of the hydroxides and silicates used for the activation process. The sustainability part comes in from the reuse of the waste which would have otherwise been landfilled and leached into groundwater. Basri et al. [124] observed that an RHA/AA range of 0.7–0.8 and NaOH between 12–14 M increased the geopolymer compressive strength upwards of 23 MPa. They further found that the Fourier transform infrared spectroscopy (FTIR) spectra of RHA, shown in Figure 12, had a relatively high ratio of the inverted peak height (H) and the inverted peak (AS) of Si-O-Si stretching vibration. This implies that both S23 (brittle) and S28 (ductile) samples underwent high geopolymerization, which produced a higher compressive strength.

#### 4.2.11. Palm Oil Fuel Ash

**Figure 13:**
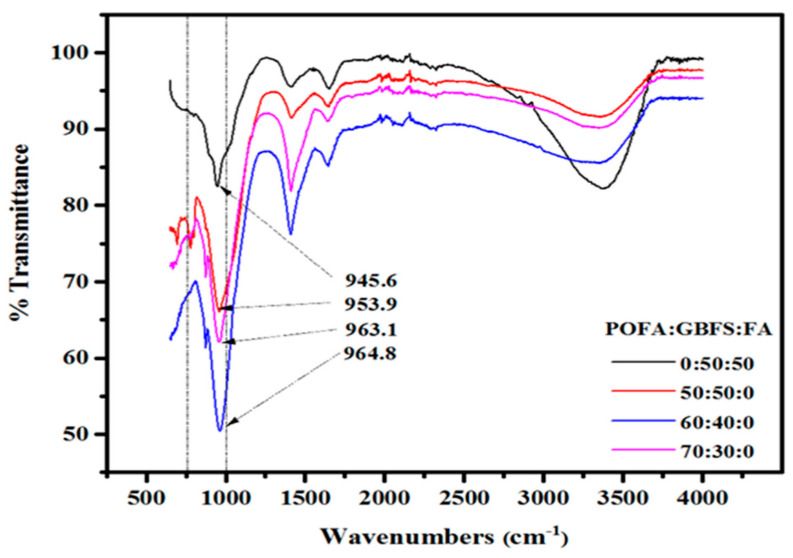
FTIR of AAMs with different POFA levels [126].

Palm oil fuel ash (POFA) is an industrial waste collected from the combustion of palm oil waste as fuel to generate electricity. Ranjbar et al. [125] studied the compressive strength and microstructural analysis of FA-POFA-based geopolymer mortar and found that the increase in POFA/FA ratio increased the SiO_2_/Al_2_O_3_ ratio leading to a decrease in early age strength and later gradual strength gains attributed to the reaction of aluminate species in early stages/scarcity of Al at later stages, and dominance in the reaction of silicate species at later stages. Similar results were obtained by [126], who attributed the loss in strength to a decrease in the dense gel formation and changes in crystallinity as the POFA replacement levels increased from 50–70% by weight of GGBFS. Figure 13 shows the FTIR of alkali-activated materials prepared with different levels of POFA. After adding POFA at 50, 60, and 70% replacement levels, the stretching vibrations of Si-O-Al emerged at 956.9, 963.1, and 964.8 cm^−1^, respectively. The mixtures showed significant structural alterations due to higher content of SiO_2_ and a decrease in C-(N)-A-S-H and C-S-H gel production leading to a decrease in mechanical strength and delayed geopolymerization process.

#### 4.2.12. Waste Glass

**Figure 14:**
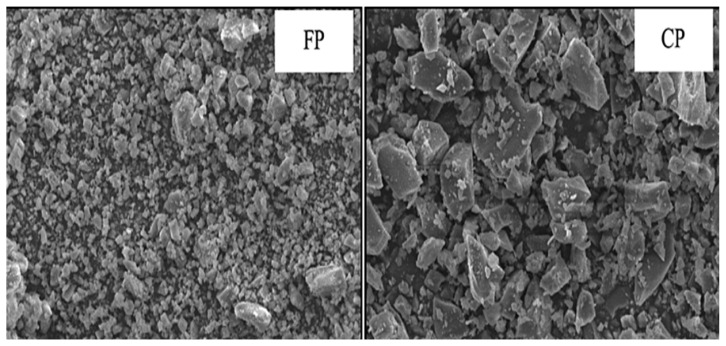
SEM image of waste glass power [128].

The manufacturing and consumption of glass have increased globally due to an advancement in the standards of living. Waste glass (WG) is non-biodegradable and non-combustible, thus leading to increased landfills. Due to its high amorphous silica content, waste glass powder has the potential to be used as a silica source just as fly ash. Xiao et al. [127] studied the strength of waste glass geopolymers cured at ambient temperature and found that the geopolymer obtained by mixing 25WG:75FA (WG/FA = 1:3) at a Si/Al ratio of 3.038 and 5 M sodium hydroxide gave a compressive strength of 34.5 Mpa making it suitable for usage as a precursor in geopolymer production. In another study by [128], waste glass powder replaced 10 to 40% of Class C fly ash to produce geopolymer paste cured at 60 °C for 48 h and then held at room temperature (23 °C) giving a 7-day compressive strength range of 34–48 Mpa. The 10–20% replacement levels by weight yielded the best optimum mechanical results. Similar results were obtained by [129], who attributed the improved compressive strength performance to reactive SiO_2_ and Al_2_O_3,_ contributing to the generation of N-A-S-H gel. Figure 14 shows the SEM images of ground fluorescent lamp glass (FP) and ground container glass (CP) powders [128] having irregular angular particles, smooth surfaces, and sharp edges.

## 5. Alkaline Activators and Their Properties

The properties of a geopolymer are highly dependent on the activator type and concentration.

### 5.1. Alkaline Type

Full activation of the aluminosilicate source/precursor can be achieved by using alkali hydroxides, silicates, sulfates, and carbonates [58]. The activator (one part or mixture) to be mixed with the precursor is usually in solution form and rarely in solid form due to the hygroscopic nature of the activator, which might cause reactivity problems [130]. The commonly available alkaline activators comprise NaOH, Na_2_SO_4_, Na_2_SiO_3_, Na_2_CO_3_, K_2_CO_3_, KOH, K_2_SO_4_, and cement clinker. The activators commonly used for geopolymer production are a mixture of NaOH or KOH and Na_2_SiO_3_ or K_2_SiO_3_ [16,131]. NaOH is a commonly used alkaline activator and its effectiveness in the geopolymerization process is dependent on its molar concentration [132,133]. The usage of sodium hydroxide leads to high-early strength gains, but lower late-strength gains as compared to sodium silicate. Sodium silicate is a combination of sodium oxide (Na_2_O) and silicate (SiO_2_) with some water, having the general formula Na_2_O.nSiO_2,_ where n refers to the modulus of silicate defining the number of moles of SiO_2_ per mole of Na_2_O [131]. The different manufacturing methods (e.g., hydrothermal and alkaline fusion) lead to the production of Na_2_SiO_3_ with different properties in terms of silicate modulus and solid/water ratio. For geopolymer production, the commonly used Na_2_SiO_3_ has a silicate modulus of 2 to 3.3 and solid content of 37 to 48 wt.% [131,134]. The usage of sodium silicate as an alkali activator gives the highest compressive strength but leads to fast setting and high-drying shrinkage, which can be solved by extending the time of mixing [130].

According to [131], KOH possesses a high potential for dissolution of aluminosilicate due to its high alkalinity compared to NaOH. However, the utilization of either NaOH or KOH eventually can form silicate and aluminate monomers. The mixing of sodium hydroxide with sodium silicate leads to a stronger geopolymerization due to the additional soluble silicate (SiO_2_) and alkali content (Na_2_O) that increases the SiO_2_/Na_2_O in the activator [16,79]. The choice of an alkali activator for geopolymer production impacts the economy and the environment. The utilization of NaOH produces less CO_2_ emission compared to Na_2_SiO_3,_ which requires higher calcination temperatures for Na_2_CO_3_ and SiO_2_. Therefore, the large-scale utilization of commercially produced sodium silicate as an activator will meet limitations in terms of scalability, cost, energy demand, practical handling issues, and the environmental cost of the product [135]; however, the benefits of using geopolymer concrete offset its limitations as compared to ordinary Portland cement concrete mainly through waste materials reutilization, supporting the circular economy, lower precursor cost, lower CO_2_ emission, higher thermal resistance, superior mechanical performance, acid resistance, sulfate attack resistance, lower drying shrinkage, better frost resistance, and dimensional stability [130,136,137]. Luukkonen et al. [138] reported on the state-of-the-art of one-part alkali-activated materials. They observed that the development of one part or “just add water” alkali-activated materials have greater potential for in situ applications as compared to conventional two-part alkali-activated materials. Askarian et al. [139] evaluated the mix composition and characterization of one-part geopolymers with different activators to replace highly corrosive alkali solutions used for geopolymer production. 100% fly ash or a mixture of 50% fly ash + 50% slag was activated solely with Na_2_SiO_3_, Ca(OH)_2_, K_2_CO_3_, LiOH, and Na_2_O in powder form. They found that Na_2_SiO_3_ used as a sole activator gave a weak porous structure with low geopolymer strength, Ca(OH)_2_ gave an increased compressive strength, but lower workability, Na_2_SiO_3_ + Ca(OH)_2_ + K_2_CO_3_ gave low strength and workability, Na_2_SiO_3_ + Ca(OH)_2_ + LiOH/Na_2_O gave a relatively high compressive strength. A similar study on one part or “just add water” alkali-activated materials was conducted by [140], who found that high strength one part alkali-activated materials can be achieved by the activation of fly ash with KOH + anhydrous sodium metasilicate at room temperature.

### 5.2. Alkaline Concentration

**Figure 15:**
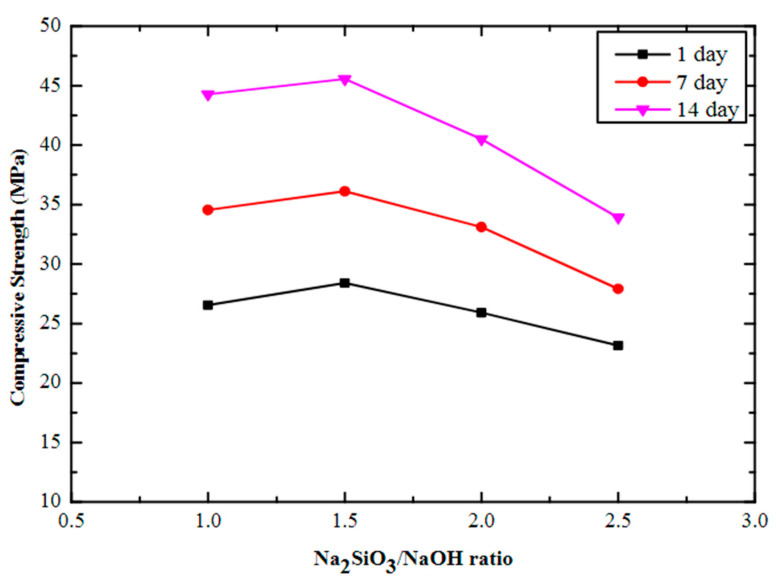
Variation effect of Na_2_SiO_3_/NaOH ratio on compressive strength [133].

The nature of the aluminosilicate source, alkali activator, and curing condition strongly influences the optimal dosage and concentration. An increase in the curing temperature and silica/alumina ratio increases the compressive strength [135]. In addition, the liquid/binder ratio has a dominant role in the development of geopolymer strength. According to a study by [132], the compressive strength of fly ash-based geopolymer concrete increased when the NaOH molarity increased from 8M to 16M. However, raising the NaOH above 16M led to a decrease in compressive strength. Similar results were obtained by [141]. The setting time of geopolymer depends on the dissolution rate of the aluminosilicate source and the precipitation of the reaction products [130]. The activator type plus concentration influences the behavior of geopolymers. Morsy et al. [16] studied the effect of sodium silicate to sodium hydroxide ratios (0.5, 1.0, 1.5, 2.0, and 2.5) on the strength of fly ash geopolymer binder at 80 °C curing temperature. The NaOH pellets with 97% purity were mixed with Na_2_SiO_3_ (composition of Na_2_O = 7.9%, SiO_2_ = 26.0%, and H_2_O = 66.1%, and Ms (SiO_2_/Na_2_O) = 3.29) to create the alkaline activator. They found that the compressive strength increased as the sodium silicate/sodium hydroxide ratio increased from 0.5 to 1.0 attributed to a homogeneous and less porous microstructure formation. The compressive strength then decreased as the sodium silicate/sodium hydroxide ratio increased from 1.0 to 2.5 attributed to excess Na_2_SiO_3_ hindering water evaporation and structure formation. Contrary results were found by [133], who reported the effect of Na_2_SiO_3_/NaOH ratio (1, 1.5, 2, and 2.5) and NaOH molarity (10M, 12M, 14M, 16M, and 18M) on the synthesis of fly-ash based geopolymer mortar at 70 °C curing temperature. The NaOH pellets with 98% purity dissolved in distilled water were mixed with Na_2_SiO_3_ solution (composition of Na_2_O = 11%, SiO_2_ = 31.7%, and H_2_O = 57.3%, and Ms = 2.88) to create the alkaline activator. Figure 15 shows the effect of varying the Na_2_SiO_3_/NaOH ratio on compressive strength. They observed that an increase in Na_2_SiO_3_/NaOH ratio from 1 to 1.5 and NaOH from 10M to 16M led to an increase in compressive strength and thereafter, beyond 1.5 Na_2_SiO_3_/NaOH ratio and 16M NaOH, the compressive strength decreased. The increase in strength up to the 1.5 ratio can be attributed to the increase in soluble silicates, which assist in the polycondensation of the geopolymeric matrix, while the decrease in strength beyond the 1.5 ratio can be attributed to excess soluble silicates hindering the formation of geopolymeric matrix. The increase in strength up to 16M is attributed to enhanced dissolution of precursor material into silicates and aluminates, while the decrease in strength beyond 16M is due to excess NaOH, which leads to rapid precipitation and hardening of the aluminosilicate gel.

Ref. [105] studied the effect of NaOH and Na_2_SiO_3_ solutions on compressive and shear bond strengths of FA-GGBFS geopolymer at 23 °C ambient curing temperature and a Na_2_SiO_3_/NaOH ratio of 2.0. The 10M NaOH solution was mixed with Na_2_SiO_3_ solution (composition of Na_2_O = 11.67%, SiO_2_ = 28.66%, and H_2_O = 59.67%, and Ms = 2.46) to create the alkaline activator. They found that for the FA and FA + GGBFS pastes, the use of NaOH solution or Na_2_SiO_3_ solution alone gave low strengths, whilst the use of NaOH + Na_2_SiO_3_ solutions gave higher strengths. Furthermore, the use of NaOH and NaOH + Na_2_SiO_3_ solutions resulted in crystalline C-S-H and amorphous gel, whilst the use of Na_2_SiO_3_ solution resulted in mainly amorphous products. In another study, Ref. [142] reported the effect of high-temperature heat treatment on alkali attack resistance of hardened fly ash geopolymer binders using a combined NaOH + Na_2_SiO_3_ solutions at 80 °C curing temperature and Na_2_SiO_3_/NaOH ratio of 2.0. The NaOH pellets of technical grade with 99% purity dissolved in distilled water to obtain 12M solution was mixed with Na_2_SiO_3_ solution (composition of Na_2_O = 8.3%, SiO_2_ = 27.7%, and H_2_O = 64%, and Ms (SiO_2_/Na_2_O) = 3.33) to create the alkaline activator. They found that exposure of hardened fly ash geopolymer mortars to high-temperature heat treatment reduced the compressive strength by 26.7% (at 200 °C) and by 66.7% (at 600 °C), partially reducing expansion and leaching due to alkali attack.

## 6. Impact of Curing

As compared to ordinary Portland cement concrete, which undergoes water curing to gain strength, geopolymer concrete undergoes heat curing (oven/steam) and ambient curing to activate the polymerization process. Curing temperature, curing time, and liquid/binder ratio affect not only the mechanical strength development but also the shrinkage and microstructure of geopolymers [137,143,144]. According to [144], the compressive strength of geopolymer mortar increased with an increase in curing temperature, a decrease in curing duration, and a decrease in delay time before heat curing. They observed that temperatures greater than 90 °C reduced the compressive strength due to the specimen’s moisture loss, thus deteriorating the strength. The heat is considered an energy booster to kickstart the geopolymer activation process, which is rather slow at room temperature. This is likely to affect the use of geopolymer under in situ conditions as it will take longer to achieve the required strength. The compressive strength at ambient temperature was too low compared to oven curing. Heat-cured geopolymer develops better mechanical strength as compared to ambient-cured geopolymer. According to [144], the optimum heat curing temperatures commonly used to facilitate the geopolymerization process and to achieve almost perfect geopolymerization range from 40 °C to 90 °C and need not be more than 24 h in practical applications. An increase in the curing duration from 24 h to 72 h decreased the compressive strength from 31.46MPa to 29.67MPa attributed to the weakening of the microstructure [144]. Contrary results were found by [42], who suggested a curing duration of three days at 40 °C and one day at 80 °C to obtain a better geopolymer network and minimize shrinkage. Authors [145,146] explored the feasibility of microwave curing and found that it enhanced the geopolymerization process and increased the compressive strength of geopolymer up to optimal levels. Ref. [90] developed a high-strength fly ash-based geopolymer in a short time by using microwave curing. In the study, sodium silicate (SS) with a SiO_2_/Na_2_O ratio of 2, and 8 M sodium hydroxide (SH) were mixed to a SS/SH weight ratio of 2.5 after which microwave curing between 5 and 105 min was performed, and compared to oven curing at 60 °C and 90 °C. Compressive strengths of 70 MPa, 77 MPa, and 90 MPa for microwave-cured specimens and compressive strengths of 44 MPa, 58 MPa, and 66 MPa for oven-cured were recorded for 30 min, 45 min, and 60 min curing times, respectively. The higher compressive strength in microwave curing is attributed to rapid and uniform heating, which accelerated the strength gain and enhanced the specimen microstructure. Ref. [92] reported the effect of slag, silica fume, and metakaolin on the properties and performance of alkali-activated fly ash cured at ambient temperature. The results showed that the inclusion of slag (S) and metakaolin (MK) improved the mechanical properties, whilst the inclusion of silica fume (SF) decreased the mechanical properties and increased the volumetric percentage of large capillary pores. The maximum 28-day compressive strength was achieved at FA100, FA85:S15, FA95:SF5, and FA90:MK10, giving 35 MPa, 47 MPa, 30 MPa, and 38 MPa, respectively. Ref. [147] studied the performance of ambient and heat-cured geopolymer composites. They found that 1-day heat cured geopolymer mortar gained more than twice the compressive strength of 28 days ambient cured geopolymer mortar. However, strength development with age was more pronounced in ambient cured geopolymer compared to heat cured geopolymer attributed to the presence of moisture in the alkali solution, which slowly reacted with the unreacted fly ash particles.

## 7. Admixture/Additive

Various experiments reported in the literature have widely utilized various admixtures/additives for the improvement of geopolymer physicomechanical performance through pore structure densification, mesopores/total porosity reduction, and crystalline phase generation [53]. Examples of mineral admixtures [148] consist of ordinary Portland cement, nano-silica, and alccofine, while chemical admixtures [149] consist of calcium chloride, calcium sulfate, sodium sulfate, and sucrose. The chemical additives [53] consist of quick lime, polyethylene glycol, cross-linked poly acrylic acid, carbon nanotubes, latex, and biofilm. The commonly used fibers [93] comprise asbestos, glass, and polypropene. Bio-additives [150], such as molasses, palm jaggery, honey, and Terminalia chebula, have also been used to improve the performance of geopolymers.

## 8. Mix Design

**Figure 16:**
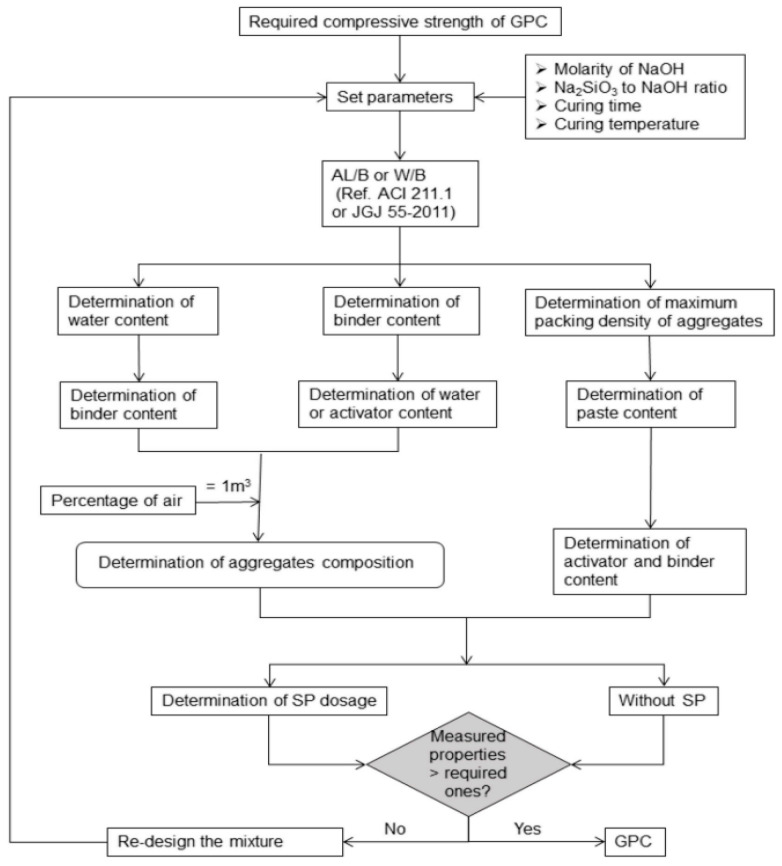
An illustration of the target strength mix design approach for geopolymer concrete [161].

Researchers are yet to develop a mix design method that is universal for geopolymer production due to the complexity of mixture proportioning variables and several factors influencing the properties of geopolymer. Currently, there are no mix design standard codes for geopolymers as most designs depend on the trial-and-error approach because of the multiple and varying mix design variables. Besides the trial-and-error approach, other researchers have applied the Taguchi approach [151,152,153,154], particle packing fraction method [155,156,157], and response surface methodology [158,159,160] for optimum mix design of geopolymer to obtain the target strength applicable only to specific materials, activator dosage, and curing conditions. The strategy adopted by researchers for geopolymer mix design has been limiting and does not represent the broad range of key variables influencing geopolymer performance properties. The adopted mix designs require the optimization of certain mixture variables whilst keeping constant the other nominal material factors consisting of aggregate grading, aggregate to binder ratio, source and type of raw material, the chemical composition of raw material, and mixing temperature. The variation in the precursor material properties hinders the development of a universal mix design. The proposition of a practical mix design requires the selection of different input and output parameters. Figure 16 shows an illustration of the target strength mix design approach for geopolymer concrete production. The main targets used to determine precursor, activator, water, and aggregate content are compressive strength and workability. In ordinary Portland cement concrete, the compressive strength is adjusted by binder content and water content.

For OPC, the mix design methods are dependent on water to cement ratio (*w/c*) and the physicomechanical performance properties. The incorporation of geopolymer as an alternative to ordinary Portland cement in the architecture, engineering, and construction (AEC) industry can help certify its viability in the long term. Lahoti et al. [162] evaluated the importance of four typical mix design parameters, consisting of water/solids, Si/Al, Al/Na, and H_2_O/Na_2_O, in determining the compressive strength of metakaolin-based geopolymers through experiments and statistical analyses. The results showed that Si/Al and Al/Na ratios are the most significant parameters governing the strength of metakaolin-based geopolymers. However, the method is limited as it does not consider the SS/SH, NaOH concentration, and compositional properties of Na_2_SiO_3_. In another study, Beulah et al. [163] observed that, depending on mix compositions, the compressive strength was dependent on Al/Si, Ca/Si, and Ca/(Si+Al) as key mix parameters. They further found that applying correlation coefficient and linear regression analysis helped to empirically capture the complexity of geopolymerization. Machine learning-based classifiers predicted the compressive strength with high precision, guiding the preliminary mix proportioning to achieve the required strength grade without the tedious trial-and-error mix formulations; however, not all mixture variables were considered for the mix design, thereby limiting its applicability to FA-GGBFS and 6 M/12 M NaOH + Na_2_SiO_3_. Further, the aggregate type and grading, quantity, and fineness of raw material, SS/SH, and compositional properties of Na_2_SiO_3_ were not considered. Ref. [80] modeled and optimized fly ash-slag-based geopolymer using the response surface method. The main experimental independent variables chosen for the investigation of the mechanical performance of soft soil were alkali equivalent (range of 6–10%), activator modulus (range of 0.8–1.2), and slag replacement ratio (range of 0.2–0.4). In the study, the optimized conditions of 9.988% alkali equivalent, 1.030 activator modulus, and 0.328 slag replacement ratio led to the global desirability of the predicted strength values of 3 days (28.19 MPa) and 28 days (54.69 MPa). These results showed that geopolymer can be used in soil stabilization satisfactorily, thereby mitigating the environmental concerns caused by ordinary Portland cement.

However, a mix design method that does not include the influence of all the compositional properties of alkaline activators limits its applicability in industry. None of the proposed mix design methods in the reviewed literature has considered the inclusion of all alkaline activator parameters, precursor, and aggregate as key variables.

## 9. Microstructure

**Figure 17:**
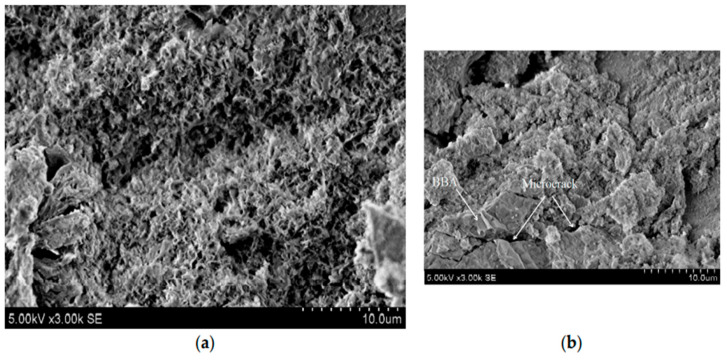
Microstructure of alkali-activated biomass bottom ash-phosphogypsum samples [98]. (**a**) PG 15-3; (**b**) PGWG 15-3.

The physico-chemo-mechanical properties of a geopolymer vary depending on the utilized raw material hence the need to understand the precursor and microstructural response to the activator solution [40]. Vaiciukyniene et al. [98] pointed out that the substitution of biomass bottom ash with 15% phosphogypsum at a SiO_2_/Na_2_O molar ratio of 3 in NaOH activating solution (PG 15-3) and the substitution of biomass bottom ash with 15% phosphogypsum at a SiO_2_/Na_2_O molar ratio of 3 in NaOH + Na_2_SiO_3_ activating solution (PGWG 15-3) exhibited different microstructures after 28 days of hardening. Figure 17a shows that the microstructure of the PG15-3 sample had a varied honeycomb-like C-S-H and amorphous gel structure. Figure 17b shows that the PGWG 15-3 had a more compact microstructure closely related to the increased amount of hydration products (C-S-H and AFt). The AFt increased the number of microcracks and expansion of geopolymer. Similar results were found by [93], who attributed the expansion and crack development to ettringite formation at a later age and hence recommended the need to study the phosphogypsum-slag hydration and crack formation mechanism.

Gijbels et al. [164] found that the incorporation of phosphogypsum up to an optimum level gives rise to C-A-S-H type gel and a higher polymerized network with a more compact, close texture, interlocking arrangement microstructure that leads to enhanced compressive strength. Ref. [100] observed that the decrease in compressive strength beyond an optimum level of phosphogypsum was related to the un-reacted phosphogypsum acting as a barrier against geopolymer chain formation, which led to the reduction in the microstructure cohesion.

## 10. Mechanical Properties

Mahdi et al. [165] observed that the addition of brick kiln rice husk ash to fly ash geopolymer paver blocks led to an increase in abrasion resistance as well as an increase in compressive, splitting tensile, and flexural strength of the fly ash-brick kiln rice husk ash geopolymer paver blocks. This was attributed to the improvement of the microstructure, having a stronger bond between polymeric gel and aggregates, making it denser and compact. Ref. [166] observed a linear relationship between compressive strength, splitting tensile strength, and bond strength of geopolymer concrete, attributing it to good interfacial transition zone (ITZ) characteristics and the presence of calcium in the geopolymer matrix. Song et al. [167] reported that the addition of steel slag (up to 20% by weight) on high calcium fly ash geopolymer pastes exhibited a lower sorptivity coefficient, higher compressive strength, and a higher elasticity modulus which was attributed to a denser and stronger geopolymeric matrix of C-S-H and C-A-S-H gels. Similar results were observed by Part et al. [168], who concluded that blended waste geopolymer mixtures improve the setting times, microstructure, and strength development of geopolymers through the alteration of the silica/alumina (Si/Al) ratio, and calcium/silica (Ca/Si) ratio in geopolymer systems. The utilization of high calcium industrial by-products such as ground granulated blast furnace slag [164,169], Class-C fly ash [166,170], and phosphogypsum [99,100] can give C-A-S-H gels with higher polymerized networks. This led to higher strength gains and improved setting time, which is beneficial for precast and in situ construction applications [15,168].

Yacob et al. [171] evaluated the shear strength of a Class F fly ash-based geopolymer concrete and concluded that the variation in shear span-to-effective depth ratio (a/d) from 2.0 to 2.4 changed the failure mode from shear to shear-flexure. The strain measurements confirmed the visual development of shear cracks followed by flexural cracks, where the absence of stirrups led to brittle failure once the beam reached its shear strength. Ref. [172] reported that reinforced geopolymer structural members can be used in terms of their ultimate load-carrying capacity and the standard code of practice for conventional concrete can be applied for geopolymer concrete structural design whilst ongoing research should develop a fit-for-purpose standard design method for reinforced geopolymer concrete structural members. Some researchers utilized ANSYS [173,174], and ABAQUS [175] software for the numerical analysis and finite element modeling of geopolymer concrete structural members, where good correlations were obtained between the experimental and simulated load-deflection behavior of reinforced geopolymer concrete beams. Furthermore, several researchers have supplemented and validated the mechanical laboratory tests with machine learning techniques to predict and model the geopolymer compressive strength [176,177,178,179]. Compressive strength is the most important mechanical parameter as it strongly affects the concrete element’s safety and durability [180].

## 11. Durability

Durability broadly refers to the capacity of a material to withstand the action of weathering, chemical attack (e.g., alkali–silica reaction, carbonation, chloride attack, sulfate attack, acid attack), physical attack (e.g., freeze–thaw, high temperature), mechanical attack (e.g., abrasion, erosion, cavitation), and constructional defects (e.g., improper consolidation and curing) whilst maintaining its performance throughout its service life. Albitar et al. [181] reported the durability evaluation of geopolymer and conventional concrete exposed to 5% sodium chloride, 5% sodium sulphate, 5% sodium sulphate + 5% magnesium sulphate, and 3% sulphuric acid. Overall, the study concluded that geopolymer concrete exhibited superior durability performance (compressive strength, flexural strength, splitting tensile strength, and weight changes) under chemical attack but higher water absorption and sorptivity rate as compared to OPC concrete. However, the addition of graphene nanoplatelets to geopolymer binders enhances porosity, water absorption, and sorptivity [182]. Naghizadeh et al. [89] reported that the severity of alkali attack on the physicomechanical properties (compressive strength, expansion, and weight change) of fly ash geopolymer binder increased with an increase in sodium hydroxide media concentration (exceeding 1M) and in storage temperature (23, 38, and 80 °C) where new phases of crystallinity comprising of phillipsite, zeolite-P, and chabazite formed. Aiken et al. [91] studied the resistance of fly ash geopolymer binders to organic acids following exposure to acetic and lactic acid. The study results showed that the surface of fly ash-based geopolymers had superior resistance to organic acids compared to ordinary Portland cement.

Rashad [100] studied the durability of alkali-activated fly ash (FA) calcined phosphogypsum (CPG) paste under elevated temperatures (400, 600, 800, and 1000 °C) and thermal shock through water quenching. It was found that the residual strength for all mixtures increased with increasing heat treatments with CPG5FA95 paste having the highest resistance to elevated temperatures and CPG0FA100 having the highest thermal shock resistance. Naghizadeh et al. [142] subjected hardened geopolymer mortars to high-temperature heat treatment (100, 200, 400, and 600 °C) for 6 h in different media solutions comprising water, 1M, and 3M sodium hydroxide and stored at 80 °C for 28 days. The results showed that the geopolymer mortars subjected to 100, 200, 400, and 600 °C and stored in 3 M sodium hydroxide reduced the compressive strength by 78%, 61%, 74%, and 82% in compressive strength, respectively, relative to the control sample. This severe reduction in strength is attributed to the release of Si and Al from the aluminosilicate gel network under alkali attack. It was therefore concluded that the temperature of 200 °C gave the least expansion and reduction in compressive strength under alkali attack. However, there is a greater need for further research on the durability of geopolymers relative to ordinary Portland cement to facilitate standards development and full technology adoption [57,59,183,184].

## 12. Cost–Benefit

**Figure 18:**
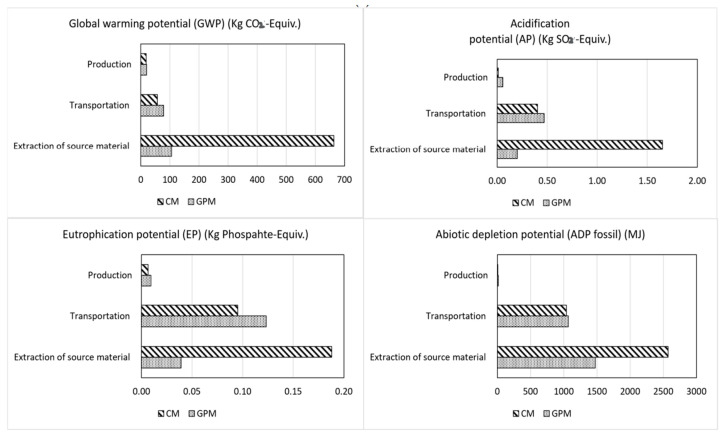
Life cycle assessment for geopolymer and OPC mortar [147].

Abdollahnejad et al. [185] studied the cost analysis of fly ash-based geopolymer foam and found that the forming agents (hydrogen peroxide and sodium perborate) are responsible for less than 10% of the total cost whilst the alkaline activators (sodium hydroxide and sodium silicate) are responsible for more than 80% of the total cost. The lower the activator/binder ratio, the lower the cost. Therefore, the replacement of sodium silicate with low-cost waste glass [185], silica fume [186], and rice husk ash [70] whilst retaining comparable mechanical strength can make geopolymer manufacturing cost-efficient. Nevertheless, the usage of geopolymer as compared to ordinary Portland cement brings about benefits in sustainability [187,188], lower CO_2_-e emissions [1,25], lower production cost [186], and comparable mechanical properties [58], offsetting the initial material cost and energy requirements. However, to increase practicality, additional research still needs to be performed on the development of cost-effective low-energy activators and curing at low/ambient temperature [35]. Furthermore, CO_2_ abatement, sustainability, and technical benefits over cost reduction are now considered highly in the development of alternative cement [34]. The production of geopolymer mortar is more environmentally friendly compared to OPC mortar. For example, Figure 18 shows that 1 m^3^ of geopolymer mortar produces 72% GWP, 65% AP, 41% EP, and 29% ADP less than the production of 1 m^3^ of OPC [147]. The main processes studied were the extraction of source materials, transportation of the material, and the production of the final product. The major environmental impact for geopolymer is related to the high energy consumed during the production of the alkaline activators, whilst that for OPC is related to emissions from the calcination of limestone and the combustion of fossil fuels.

The production cost of Na_2_SO_3_ and NaOH, and the selling price of geopolymer affect the economic feasibility of large-scale geopolymer production [189]. The utilization of locally available materials for geopolymer production can bring about significant savings in transportation costs and energy demand as compared to ordinary Portland cement [25,188]. The —cost-benefit results can help investors and policymakers to be aware of the practicality of implementing geopolymer production.

## 13. Applications

**Figure 19:**
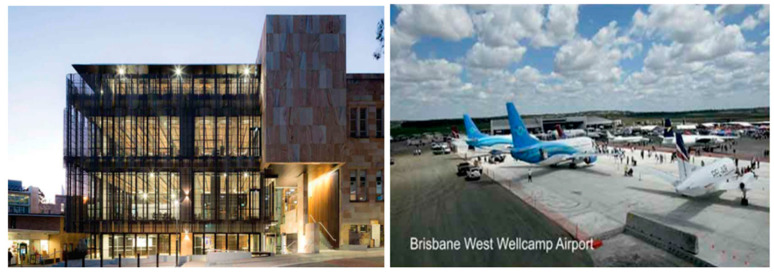
Global change institute building and Brisbane airport [12].

Geopolymer provides an alternative construction material to OPC through the reuse of industrial by-products and waste materials, thereby addressing landfill environmental impact. Geopolymers have found applications in areas of fire-resistant materials, novel ceramics, asbestos-free materials, low-tech building materials, hazardous waste stabilization, adsorbent material for water treatment, etc. [190]. Cement and concrete are the backbones of infrastructural development and modern civilization. Geopolymers are regarded as an alternative route for the construction of infrastructure in relation to the geographical area and supply chains of precursor material [46]. Fly ash-based geopolymer concrete has been successfully utilized in the construction of the University of Queensland Global Change Institute Building (Figure 19) in Australia, where 33 no. 11m span precast floor beams made of geopolymer concrete (about 320 m^3^) formed three suspended floors in the building. The Brisbane West Wellcamp Airport (Figure 19) in Australia used about 40,000 m^3^ (100,000 tonnes) of geopolymer concrete and saved 6600 tonnes of CO_2_ emission [12].

In South Africa, fly ash-slag geopolymer concrete was used to construct a concrete slab at the City Deep container terminal, achieving a compressive strength of 51MPa in 28 days [191]. However, the major issues still hindering the full adoption of geopolymer in the industry consist of alkaline activator aggressiveness, high temperature curing conditions, mix design complexity, precursor materials diversity and supply chain constraints, and the absence of long-term durability studies just as OPC [58,184]. Nevertheless, industry players, researchers, and regulatory agencies have shown interest in the innovation and value of the commercialization and large-scale production potential of geopolymers.

## 14. Limitations of the Review

Despite the significance of this review, it has its limitations. The methodology of the review is data-driven and hence dependent on the quality of data collected from databases. Even though data were collected through bibliometric techniques, the keywords combination of “geopolymer”, “geopolymer mortar”, “geopolymer concrete” and “alkali-activated materials” does not guarantee retrieval of alternate keywords such as “green concrete”, “geocement” adopted by other authors. This study was limited to the Scopus database due to its wider coverage as compared to the Web of Science and Google Scholar databases. With the exclusion of non-English documents, the search results might be limited and are likely to increase with their inclusion. These limitations could serve as further research areas for systematic literature reviews on similar studies. Despite the limitations, a remarkable change to the present review is not expected.

## 15. Future Research Areas

The following gaps were identified through the review and require further studies:

i. Presently, there is limited research on the physico-chemo-mechanical performance and behavior of a binary system of geopolymerized fly ash-phosphogypsum binder mortar at ambient curing conditions. There is a need to fully understand the hydration mechanism, crack mechanism, volumetric phase assemblages, failure mode, and resistance to physical and chemical attacks.
ii. Further studies are required to fully understand the performance of reclaimed fly ash for geopolymer production. The development of techniques to extract silica from coal ash tailings is a potential source of creating value from waste.
iii. The development of alternative activators to hydroxides and silicates with less environmental impact and minimal cost. The production of alkaline activators produces CO_2_ via the electrolysis of salts; therefore, there is a need for new bio-materials to replace the existing alkaline activators.
iv. The utilization of a one-part activator is a research route that seems promising and needs long-term performance studies. There is a need to understand the influence on microstructure development, dosage optimization, and mechanical strength. Just like ordinary Portland cement, the development of “just add” water geopolymer is still a novel area that requires further study.
v. Most research is limited to high-temperature heat curing conditions, which are expensive and energy intensive. Therefore, research on low-temperature ambient curing conditions should be performed to reduce energy requirements. The level of acceptance of geopolymers can be expanded if they can be sustainably and economically produced at low energy and cost.
vi. The use of in situ curing requires materials that should not only reduce the setting time but also improve the early age strength. The correlation between setting time and early age strength gain of geopolymer at ambient temperature needs further study.
vii. Long-term repetitive tests under different laboratory and in situ testing conditions are required to comprehensively define the durability and microstructural properties of geopolymers to facilitate the development of test methods and validation techniques. Additional long-term curing studies for geopolymer are needed since the 28-day curing regime was designed for ordinary Portland cement concrete.
viii. There is very limited research on the prediction and optimization of geopolymer concrete strength concerning mix design parameters such as precursor properties, Si/Al ratio, Ca/(Si+Al), water/solid ratio, Al/Na_2_O ratio, H_2_O/Na_2_O ratio, and curing conditions. There is a need to develop a harmonized fit-for-purpose mix design standard that considers all geopolymer production variables.
ix. There is a need to understand the shear strength and stress-strain behavior of geopolymer structural members to increase industrial application.

## 16. Conclusions

This review provided clarity on the current state-of-the-art geopolymer definitions and mix design methodologies. Geopolymer is defined as an inorganic aluminosilicate polymer product from the chemical reaction of amorphous aluminosilicate geomaterials and alkaline activating solutions. This alternative construction material has the potential to reduce the pressure on the environment and conserve natural resources. The utilization of geopolymer as an alternative to ordinary Portland cement for construction reduces the overall CO_2_ emission by 9% to 64%, dependent on the geographic location, transport scenarios, alkali activator point of sale and manufacture, and mix design basis. Geopolymer properties are dependent on the precursor, activator type and concentration, mix design, and curing condition. The physical properties of the precursor consisting of particle size distribution, specific surface area, and specific gravity, influence the reactivity of the precursor in tandem with strength. Finer-sized precursors are better since they increase the surface area upon which the alkaline activator can dissolute the aluminosilicate. The major functional groups present in geopolymer consist of Si and Al. A range of NaOH molarity from 5 to 16M, Na_2_SiO_3_/NaOH from 0.5 to 2.5, SiO_2_/Na_2_O from 0.7 to 5, Si/Al from 0.75 to 6, Ca/Si from 0.107 to 0.401, and liquid/binder ratio less than 0.35 have been used for making different geopolymer formulations. Compared to ambient curing, heat curing temperatures ranging from 40–90 °C and curing time from 24–72 h achieve better geopolymerization and give better strength output. Blended waste geopolymer mixtures improve the performance of geopolymers through the alteration of the Si/Al ratio and Ca/Si ratio in the geopolymeric matrix. Besides the blending of fly ash with other industrial by-products and waste materials, phosphogypsum also has the potential for use as an ingredient for unary and binary geopolymer production. The incorporation of phosphogypsum, up to 15% replacement levels, has a favorable impact on the setting time, Ca/Si ratio, C-A-S-H gel, and strength of geopolymer. The development of C-S-H or C-A-S-H in blended geopolymer systems densifies the microstructure and reduces permeability in aggressive environments.

As there is no geopolymer mix design standard, most of the mix designs are performed using the trial-and-error approach, while other mix designs apply the Taguchi approach, particle packing fraction method, and response surface methodology to obtain the targeted strength of geopolymer. The trial-and-error approach is the most used due to the variation in source materials and alkaline activators. The challenge with this approach is the huge amount of ingredients wasted in trying to obtain the right mix proportioning. Researchers are now much more focused on the Taguchi approach by employing the target strength technique. This is more of a reverse analysis in such a way that the mix design is performed to achieve a predetermined strength. The challenge with the mix design approaches except trial and error is that they rarely consider all the variables as input to achieve the output; hence, it might not truly represent the effectiveness of the technique; therefore, there is a need to accumulate more research data through the trial-and-error approach, which will help to correlate and develop a robust mix design. The cost of sodium hydroxide and sodium silicate, precursor availability, and geopolymer selling price are the major factors influencing the economic feasibility of geopolymer production. Overall, the use of geopolymer as compared to ordinary Portland cement brings about benefits in lower CO_2_-e emissions, lower production cost, and comparable mechanical properties offsetting the initial activator cost and energy requirements. The adoption of geopolymer by the construction industry and regulatory authorities as a “go-to” alternative to OPC will require additional long-term durability studies to obtain enough data for standards development. A less permeable and porous geopolymer product prevents the ingress of aggressive liquids into the geopolymer matrix, thereby protecting the geopolymer from physical and chemical attacks. Geopolymer is yet to attain the level of durability confidence as OPC due to the absence of robust mix design standards.

## Figures and Tables

**Table 1:** Inclusion criteria applied during data retrieval from Scopus as of 25 April 2022.

Option	Inclusion Criteria Applied
Language	English
Publication date	2011–2022 (April)
Subject area	Engineering; Material Science; Environmental Science
Source type	Journal
Document Type	Article, Review
Availability	Full text

**Table 2:** Chemical composition of fly ash in different areas of the world.

Country	Oxides (%)											
	SiO_2_	Al_2_O_3_	Fe_2_O_3_	CaO	MgO	MnO	K_2_O	Na_2_O	TiO_2_	P_2_O_5_	SO_3_	LOI
South Africa [89]	56.45	30.27	3.58	4.59	1.06	-	0.77	0.14	1.57	0.38	-	0.42
India [86]	61.16	30.48	4.62	1.75	0.18	-	0.18	0.76	1.56	0.27	0.19	0.60
China [80]	54.6	27.2	11.6	2.2	1.0	-	0.7	1.0	0.5	-	-	1.0
Australia [90]	51.11	25.56	12.48	4.3	1.45	0.15	0.7	0.77	1.32	0.885	0.24	0.57
United Kingdom [91]	46.78	22.52	9.15	2.24	1.33	0.05	4.09	0.89	1.05	-	0.90	3.57
United States of America [92]	56.52	22.75	4.56	8.53	2.64	-	1.16	0.69	-	-	0.4	0.35

**Table 3:** Chemical composition of phosphogypsum in different areas of the world.

Country	Oxides (%)												%
	SiO_2_	Al_2_O_3_	Fe_2_O_3_	CaO	MgO	MnO	K_2_O	Na_2_O	TiO_2_	P_2_O_5_	SO_3_	F	LOI
South Africa [96]	1.37	0.23	0.121	44	-	-	-	-	-	1.28	51	1.06	-
India [94]	1.75	0.13	0.16	38.87	0.02	-	-	0.11	0.05	1.04	52.94	0.32	3.97
China [93]	4.86	4.38	-	31.05	0.26	-	0.41	-	0.2	3.57	30.95	-	22.91
United Kingdom [95]	2.4	0.40	0.23	40	0.04	-	0.03	0.13	0.03	0.95	52	0.14	6.40
United States of America [95]	3	0.3	0.2	31	-	-	-	-	0.04	2.25	55	0.2	17.7

## Data Availability

Not applicable.
